# Acute acalculous cholecystitis and cardiovascular disease, which came first? After two hundred years still the classic chicken and eggs debate: A review of literature

**DOI:** 10.1016/j.amsu.2022.103668

**Published:** 2022-04-29

**Authors:** Martina Saragò, Davide Fiore, Salvatore De Rosa, Angela Amaddeo, Lucrezia Pulitanò, Cristina Bozzarello, Antonio Maria Iannello, Giuseppe Sammarco, Ciro Indolfi, Antonia Rizzuto

**Affiliations:** Department of Medical and Surgical Sciences, University Magna Græcia of Catanzaro, Italy

**Keywords:** Acute acalculous cholecystitis, Cardiovascular disease, Hypoperfusion, Laparoscopic cholecystectomy, Gallbladder disease, Cholecystostomy, AAC, acute acalculous cholecystitis, CVD, Cardiovascular disease, US, ultrasonography, AMI, Acute myocardical infarction, CT, Computer Tomography

## Abstract

The existence of a close association between disease of the biliary tract and disease of the heart is known from the mists of time.

Acute acalculous cholecystitis (AAC) can be defined as an acute necro inflammatory disease of the gallbladder in the absence of cholelithiasis.

AAC is a challenging diagnosis. The atypical clinical onset associated to a paucity and similarity of symptoms and to laboratory data mimicking cardiovascular disease (CVD) often results in under and misdiagnosed cases. Moreover, AAC has commonly a fulminant course compared to calculous cholecystitis and it is often associated with gangrene, perforation and empyema as well as considerable morbidity and mortality (up 50%). Early diagnosis is crucial to a prompt treatment in order to avoid complications and to increase survivability.

Even today, although scientific evidence dating two hundred years has shown a close association between AAC and CVD, due to the lack of RCT, there is still a lot of confusion regarding the relationship and consequently the clinical management AAC and CVD.

In addition, emergency physicians are not always familiar with transient ECG changes with AAC.

The aim of this review was to provide evidence regarding epidemiology, pathophysiology, clinical presentation and treatment of the complex association between AAC and CVD.

Our main findings indicate that AAC should be suspected after each general disease leading to hypoperfusion such as cardiovascular diseases or cerebrovascular diseases or major heart or aortic surgery. ECG changes in absence of significant laboratory data for IMA (Acute myocardial infarction) could be related to a misdiagnosed AAC.

US – Ultrasonography-plays a key role in the early diagnosis and also in the follow up of AAC.

Cholecystostomy and cholecystectomy as unique or sequential represent the two prevailing treatment options for AAC.

## Highlights

• AAC should be suspected after each general disease leading to hypoperfusion such as cardiovascular diseases or cerebrovascular diseases or major heart or aortic surgery.
• ECG changes in absence of significant laboratory data for IMA could be related to a misdiagnosed AAC.
• Cholecystectomy is an only definitive treatment for AAC.
• The first report on this theme after 20 years.

## 1 Introduction

The existence of a close association between disease of the biliary tract and disease of the heart is known from the mists of time [1].

However, the interest on the relationship between cardiovascular disease (CDV) and acute cholecystitis has received major attention only at the beginning of the Nineteen century [[1], [2], [3], [4]].

In 1924 Willius and Brown [2], analyzing eighty-six unselected consecutive necropsies on patients with proven coronary disease, found a chronic cholecystitis in 26%.

Similarly, in 1931 Schwartz and Herman [3] found evidence of cholecystitis 63% of patients affected by CVD. Conversely, Laird [4] in a series of 65 patients affected by acute gallbladder disease found evidence of cardiac disease in 77% of them.

During these years many other reports [[5], [6], [7], [8], [9], [10], [11]] showed a coexistence of cardiac disorders in patients affected by disease of the biliary tract and vice versa. Fitz -Hugh [5] and Wolferth [6] observed a Cardiac Improvement following bladder surgery and consequential relief from angina and recovery from decompensation. Some years later, Weiss [7], Hamilton [7] and Strauss [8] demonstrated the effect of gallbladder disease on electrocardiogram. Changes in the T waves in significant leads, slurring and notching of the QRS complexes and elevation or depression on ST segments were the most common noted electrocardiogram abnormalities.

A possible explanation of those clinical and autoptic observations was found to be, at that time, in the common physiopathological factors common to the two diseases, such as disturbed metabolism, obesity, diet and infection.

However, all the evidence did not support the high percentage of acute acalculous cholecystitis (AAC) found in those studies. Described for the first time in 1844 by Duncan et al. [12] AAC can be defined as acute inflammatory disease of gallbladder without evidence of gallstones.

The pathophysiology of AAC is not clear, but CVD seems to play a key role [13].

The atypical clinical onset associated to a paucity and similarity of symptoms and to laboratory data mimicking CVD results often in under and misdiagnosed cases [14]. Moreover, AAC has commonly a fulminant course compared to calculous cholecystitis and it is often associated with gangrene, perforation and empyema [15,16] as well as considerable morbidity and mortality (up 50%) [17,18]. Early diagnosis is crucial to a prompt treatment in order to avoid complications and to increase survivability [13].

Even today, although scientific evidence dating two hundred years has shown a close association between AAC and CVD, due to the lack of RCT, there is still a lot of confusion regarding the relationship and consequently the clinical management of acalculous biliary disease and cardiac disease.

In addition, emergency physicians are not always familiar with transient ECG changes with AAC.

The aim of this review is to provide evidence regarding epidemiology, pathophysiology, clinical presentation and treatment of the complex association between AAC and CVD.

## 2 Materials and methods

### 2.1 Search strategy

**Fig. 1:**
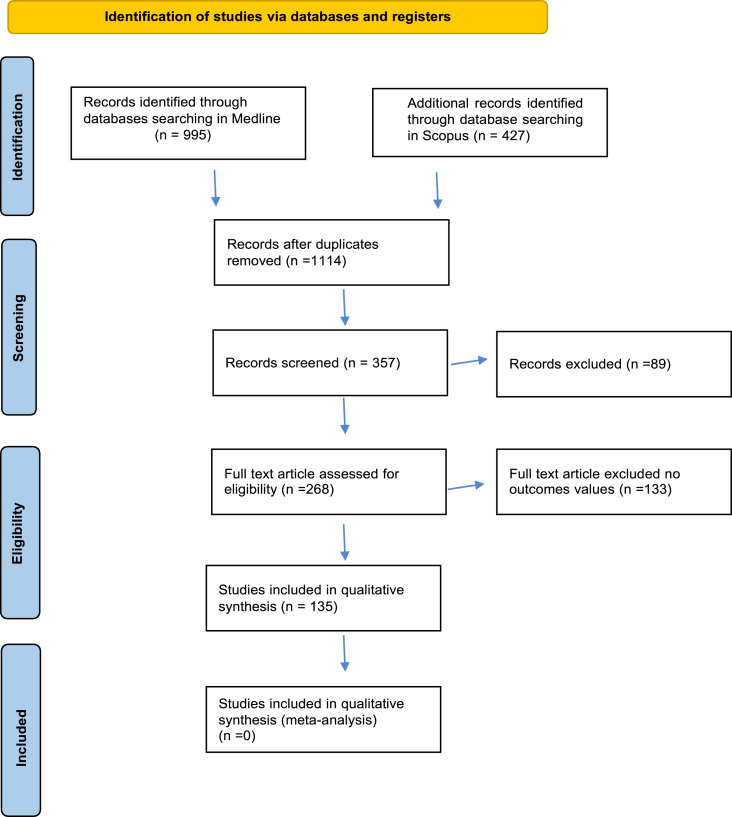
PRISMA 2020 flow diagram for new systematic reviews which included searches of databases and registers only.

We searched for publications addressing Acalculous cholecystitis and cardiovascular disease, consulting Medline and Scopus databases. Any retrospective or prospective study design or systematic review focusing on the aforementioned topic and written in the English language was accepted (see full search strategy in Fig. 1) [21].

### 2.2 Study selection and quality assessment

Two reviewers judged titles and abstracts of studies for eligibility independently. Suitable articles that matched the predefined selection criteria were then obtained in full. This study was conducted and is reported in accordance with the PRISMA [88] and AMSTAR Guidelines and in accordance with the declaration of Helsinki (reviewregistry1299) [89].

## 3 Results

### 3.1 Study descriptions and inclusion

Our search rendered 1422 hits (995 from Medline and 427 from Scopus). After removing the duplicates, there were 114 studies. After progressive screening, 268 full texts were assessed for eligibility, and 135 studies were included in qualitative synthesis as shown in Fig. 1.

### 3.2 Definition

Acute acalculous cholecystitis can be defined as an acute necro inflammatory disease of the gallbladder in the absence of cholelithiasis [13,14].

### 3.3 Epidemiology

AAC is a life-threatening condition. The incidence varies from 2% to 15% between all acute cholecystitis [14,22]. Conversely to calculous cholecystitis it occurs more frequently in male about 60 years of age [22]. AAC arises in about 02%–04% of all critically ill patients [23,24]. Mortality varies from 10 to 90% (average of 30%) and is directly related to the time of diagnosis [14,19,20,[25], [26], [27], [28]].

Sessions et al. [29], in a study of 22 cases of AAC after open-heart surgery, reported a mortality rate of 32%; with a 41% of gangrenous gallbladders. Hagino et al. found a mortality rate of 71% in ACC following aortic surgery [30].

A delayed diagnosis results in higher complications rate and consequently in a fast-clinical deterioration [13].

### 3.4 Risk factors

As summarized by Owen and Jain [22], associated risk factors for the development of AAC are found to be trauma, recent surgery, shock of any kind, burn, sepsis, bacterial infections, fungal infection, parasitic infection, viral infection, critical illness, TPN, prolonged fasting, hypovolemia, post endoscopic retrograde cholangiopancreatography, increased length of hospital stay, immunodeficiency, chronic illness, vasculitis, obstruction.

### 3.5 Pathophysiology

AAC has multifactorial pathogenesis [13,14,22]. Regardless of the underlying etiology, the physiopathological results are due to visceral hypoperfusion, ischemia and reperfusion injury as well as bile stasis [[31], [32], [33], [34]].

#### 3.5.1 Role of hypotension and visceral hypoperfusion

As early as 1987 Leitman et al. [35] reported 11 cases of AAC, following cardiopulmonary bypass surgery, identifying in the hypoperfusion the beginning causes of the disease.

Similarly, many authors reported an association between hypoperfusion and organ ischemia in the development of AAC [29,[35], [36], [37], [38]]. Moneta et al. [37] in a study on patients recovering from open-heart surgery with extracorporeal circulation and prolonged bypass time, depressed cardiac output, reported 22 cases of AAC. Similarly, Hagino et al. [30] identified AAC in 6 of 7 patients following aortic reconstruction.

In the presence of arterial atherosclerosis, often found in elderly patients, the hypoperfusion leads to a worsened prognosis [25].

### 3.6 Histopathological findings

Small vessel occlusion has shown to be the predominant phenomenon in AAC.

Glenn [23,24], Becker [23], Hakala [33] and Warren [39] hypothesize a capillary thrombosis as a basic mechanism of AAC, concluding that the common cause was visceral hypoperfusion.

Conversely, Laurila J et al. [25] provided a detailed histological analysis of AAC which resulted in leukocyte margination of blood vessels, suggesting involvement of ischemia and reperfusion mediated injury. The authors noted that these types of histopathological alterations are typical of myocardium after reperfusion injury.

According to these findings, arterial occlusion in AAC could be caused by arterial wall spasm or by passive vasoconstriction due to interstitial edema. Consequently, CVD resulting in circulatory disturbances resulting in microvascular damages, could play an important role in the pathogenesis of AAC. Moreover, bile infiltration into the bladder mucosa demonstrated by many authors, validates the abnormal epithelial permeability in AAC. All this data suggests that AAC is a manifestation of systemic critical illness [40].

### 3.7 Diagnosis

The diagnosis of AAC is difficult and sometimes it seems to be elusive.

AAC is often characterized by atypical clinical onset, paucity of symptoms, overlapped with comorbidities [19,[22], [23], [24],^27^,^31^,^41^,^42^].

Moreover, there are many confounding factors such as the critical illness of patients and laboratory results may be negative or not specific at the beginning of the disease.

In particular, AAC is sometimes indistinguishable from CVD, as well as symptoms of AAC could be similar and laboratory data mimic CVD [43].

### 3.8 CVD and AAC

As mentioned above AAC and CVD are strictly correlated. AAC seems, from different studies [13,32,44,45], to be associated with each hypovolemic state, such as acute myocardial infarction (AMI) or acute heart failure (AHF). These conditions lead to consequent ischemic damage of the gallbladder.

Some authors like Sogutlu et al. or Roth et al. [46,47] reported some cases of AAC following aortic dissection Bakey type III postulating pathogenesis mechanisms as bile stasis, sepsis and ischemia.

In the same way a hypovolemic state following the treatment of some CVD itself may contribute to the pathogenesis of AAC.

Mastoraki et al. [48] reported postoperative AAC in patients who underwent open heart surgery, after exclusion of those with preoperative cholecystopathy.

### 3.9 Electrocardiogram changes and AAC

The first description of the changes in the ECG similar to those of ischemic heart disease dates back to 1878 [3].

Several studies [[49], [50], [51], [52]] have examined the changes in the ECG of biliary distention.

The most common ECG alterations noted in AAC are changes in T waves in significant leads, slurring and notching of the QRS complex and elevation or depression of the S-T segments [[49], [50], [51], [52]].

Krasna et al. [53], hypothesizing a vagally reflex mechanism, demonstrated an association between cholecystitis or biliary colic and angina pectoris, arrhythmias and non-specific ST-T waves. Seewoodhary et al. [54] associated AAC to troponinosis and rise in CPK. Conduction disturbances, Cope's sign and reflex bradycardia are reported in several series [55,56] of AAC or necrotizing cholecystitis by different authors. Furuhashi et al. [57,58] reported a Brugada-type ST segment shift secondary to acute cholecystitis.

Although a vagally mediated reflex was suggested to be responsible for a coronary vasospasm which caused the ECG abnormalities mentioned above, the real mechanism is still unknown. The theory of a cardiobiliary reflex has been supported also by several animal studies in dogs and pigs [[59], [60], [61]]. The investigators showed that the distention of the common bile duct causes decrease in coronary blood flow.

Durning SJ et al. [62] reported a resolution of ECG changes due to AAC after cholecystectomy, or antibiotic treatment within a few days.

In conclusion, at the state of art, outside these experimental observations or case series, there is no sufficient data about the incidence and distribution of ST segment/T waves changes in patients with AAC in literature.

### 3.10 Cerebrovascular disease and AAC

It has been suggested that cerebrovascular disease is a risk factor in the pathogenesis of AAC [63,64], but there is not enough evidence in medical literature regarding this subject.

Cho et al. [65] found cerebrovascular disease as an independent prognostic factor for the development of AAC. It seems that sympathetic stimulation with increased catecholamine secretion and vagal paresis caused by strong damage to the brain center could be responsible for the development of AAC in patients affected by cerebrovascular disease [66].

### 3.11 Role of imaging in the diagnosis of AAC

All considered the ultimate diagnose of AAC usually rests on imaging.

### 3.12 Ultrasonography (US)

As reported, for the first time, by Deitch and Engel [67,68], a key role in the diagnosis of AAC is certainly played by ultrasonography. Due to the possibility to allow a rapid diagnosis, the safety and absence of radiation ultrasonography- US represents the most useful diagnostic tool in the Emergency Department.

Nevertheless, the sensitivity and the specificity of US varies from 30% to 100% [19,34,41,42,[67], [68], [69], [70], [71], [72], [73]].

This large divergence comes, probably from the different criteria for sonographic diagnosis used in the mostly retrospective studies.

Several authors [13,16,41,67,68,74] suggest major criteria and minor criteria for the diagnosis of AAC.

US major diagnostic criteria are represented by wall thickness, subserosal edema or pericholecystic fluid, and intramural gas.

Hydrops and sludge are considered minor criteria.

Most studies [75,76] recommend the so-called triad composed by thickness, hydrops and sludge as the one preferred for diagnosis of AAC.

US was found to be a valuable method for early detection of AAC by Imhof et al. [77] and Raunest et al. [78] The authors highly suggested daily US in patients with initial index of suspicion of AAC and in case of gallbladder distention without signs of AAC, to follow up such patients and if other causes of illness are not found, to avoid misdiagnosis.

### 3.13 Computer tomography

CT is not suggested to offer greater benefits over US [34,79] with the addition that the patient is required to be transported.

CT is considered to be useful, by several investigators, in the diagnosis of AAC if it is still high in the differential. It shows high sensitivity and rule out other abdominal catastrophes such as mesenteric ischemia, peptic ulcer etc.

### 3.14 Treatment

Cholecystectomy and cholecystostomy (drainage of gallbladder) are the two prevailing treatments for AAC [13,18,22,42,[80], [81], [82], [83]].

Although cholecystectomy represented the definitive therapeutic option there is still a debate as to its optimal timing [23].

Cholecystostomy is suggested to be as the unique treatment or as a bridge to cholecystectomy [75,[80], [81], [82], [83]].

Boland et al. [75], somewhat too aggressively, recommended prophylactic cholecystectomy for all ICU patients with abdominal sepsis without improvement and for whom no other etiology could be identified.

Several authors [84] suggest cholecystostomy, unless strong evidence of an ischemic gallbladder exists, because of the possibility to be performed transperitoneally or transhepatically under US or CT guidance by surgeon or by interventional radiologist. The patient's condition could be optimized for surgery.

Cholecystectomy represents the definitive therapy for patients affected by AAC [85].

Laparoscopic surgery for gallbladder disease has been shown to present several advantages in comparison with standard open approach [86,87], including shorter recovery time, reduction of post-operative pain and of wound-related complications. In recent years several studies have demonstrated the safety, feasibility and effectiveness of the standard minimally invasive surgery approach for AAC.

Furthermore, laparoscopic cholecystectomy has similar morbidity and mortality compared with conventional procedure.

Either Diagnostic laparoscopy or laparotomy is strongly recommended by the Society of American Gastroenterological and Endoscopic Surgeons.

In cases of delayed diagnosis of AAC and consequently in presence of sepsis signs it is important to apply all the measures known as “Sepsis Bundle” [90], as codified since 2005 [91]. Early identification and appropriate immediate management in the initial hours after development of sepsis are shown to improve outcomes.

In conclusion, the current evidence suggests that in suspicion of AAC [80], cholecystostomy should be performed immediately. Cholecystostomy could be the definitive treatment, if the patient's condition improves with the compression (the tube can be removed after 3 weeks) or could be a bridge for a lifesaving cholecystectomy.

In dubious cases a diagnostic laparoscopy could be a possible alternative treatment.

## 4 Conclusions

AAC is a challenging diagnosis. It should be suspected after each general disease leading to hypoperfusion such as cardiovascular diseases or cerebrovascular diseases or major heart or aortic surgery. ECG changes in absence of significant laboratory data for IMA could be related to a misdiagnosed AAC.

US plays a key role in those cases in which ECG changes are the unique suspicious signs of AAC.

Cholecystostomy and cholecystectomy as unique or sequential represent the two prevailing treatment options for AAC.

Laparoscopy seems to offer several advantages in terms of surgical trauma with equal morbidity and mortality rates if compared with open surgery.

There is strong evidence for gallbladder removal.

A proper diagnosis is extremely necessary to avoid unnecessary treatments and to improve survival.

## Ethical approval

Ethical Approval was not required.

## Sources of funding

Anything to declare.

## Author contribution

**MS and DF** conceived the study, participated in its design, drafted and revised the manuscript. They equally contributed. **SD** conceived the study, participated in its design, drafted the manuscript. **AA** analysis the studies **LP** analysis the studies**CB and AMI** drafted the manuscript. **GS** analysis the data**CI** participated in the design of the study and revised the manuscript. **AR** conceived the study, conceived the design, analysed the data and revised the manuscript. All authors have read and approved the final manuscript.

## Trial registry number

None.

## Guarantor

Prof. Antonia Rizzuto is the Guarantor of the study. She conceived and conducted the study, had access to the data and controlled the decision to publish of the final manuscript previously approved by all authors.

## Provenance and peer review not commissioned

EXTERNALLY PEER-REVIEWED.

## Declaration of competing interest

The authors declare that they have no financial support, no potential conflict of interest.

## List of abbreviations

**AAC**: Acute acalculous cholecystitis
**CVD**: Cardiovascular disease
**US**: Ultrasonography
**AMI**: Acute myocardial infarction
**CT**: Computer Tomography
